# The value of Hounsfield units in predicting cage subsidence after transforaminal lumbar interbody fusion

**DOI:** 10.1186/s12891-022-05836-2

**Published:** 2022-09-22

**Authors:** Fang Xie, Zhiwei Yang, Zhipeng Tu, Peipei Huang, Zhe Wang, Zhuojing Luo, Xueyu Hu

**Affiliations:** 1 Department of Orthopaedic Surgery, The First Affiliated Hospital of Air Force Medical University, No. 127 Changle West Road, Xi’an, 710032 Shaanxi China; 2 Department of Orthopaedic Surgery, Air Force Hospital of Eastern Theater Command, No. 1 Malu Road, Nanjing, 210002 Jiangsu China

**Keywords:** Cage subsidence, Transforaminal lumbar interbody fusion, Hounsfield units, Dual x-ray absorptiometry

## Abstract

**Background:**

Cage subsidence may occur following transforaminal lumbar interbody fusion (TLIF) and lead to nonunion, foraminal height loss and other complications. Low bone quality may be a risk factor for cage subsidence. Assessing bone quality through Hounsfield units (HU) from computed tomography has been proposed in recent years. However, there is a lack of literature evaluating the correlation between HU and cage subsidence after TLIF.

**Methods:**

Two hundred and seventy-nine patients suffering from lumbar degenerative diseases from April, 2016 to August, 2018 were enrolled. All underwent one-level TLIF with a minimum of 1-year follow-up. Cage subsidence was defined as > 2 mm loss of disc height at the fusion level. The participants were divided into 2 groups: cage subsidence group (CS) and non-cage subsidence group (non-CS). Bone quality was determined by HU, bone mineral density of lumbar (BMD-l) and femoral (BMD-f) from dual-emission X-ray absorptiometry (DXA). HU of each vertebra from L1 to L4 (e.g., HU1 for HU of L1) and mean value of the four vertebrae (HUm) were calculated. Visual analog scale (VAS) of back/leg pain and Oswestry disability index (ODI) were used to report clinical outcomes.

**Results:**

Cage subsidence occurred in 82 (29.4%) cases at follow-ups. Mean age was 50.8 ± 9.0 years with a median follow-up of 18 months (range from 12 to 40 months). A total of 90.3% patients presented fusion with similar fusion rate between the two groups. ODI and VAS in leg were better in non-CS group at last follow-ups. Using receiver operating characteristic curves (ROCs) to predict cage subsidence, HUm provided a larger area under the curve (AUC) than BMD-l (Z = 3.83, *P* <  0.01) and BMD-f (Z = 2.01, *P* = 0.02). AUC for HU4 was larger than BMD-f and close to HUm (Z = 0.22, *P* = 0.481).

**Conclusions:**

Cage subsidence may indicate worse clinical outcomes. HU value could be a more effective predictor of lumbar cage subsidence compared with T-score of DXA after TLIF.

## Introduction

TLIF technique has been widely accepted as a regular method dealing with lumbar degenerative diseases since it was introduced by Harms et.al [1, 2]. Polyetheretherketone (PEEK) cages were often used in a number of lumbar fusions with satisfactory outcomes [3]. However, cage subsidence was a common complication with a rate of 32.8–54%. With the occurrence and progress of cage subsidence, the height loss of inter-vertebral and foramen space appeared, which may have a negative impact on clinical outcomes. Previous literatures reported low BMD was a potential risk factor of cage subsidence [4–6].

Some scholars considered T-score from DXA as a “golden standard” to assess bone quality in some occasions [7]. Although the method has been widely accepted, the instructive significance may be negatively influenced, as osteophyte formation and bone sclerosis could increase lumbar BMD from DXA and bone quality may be overestimated [8]. Schreiber et.al introduced Hounsfield units (HU) measured from CT to assess bone quality and the value has been utilized in predicting pedicle screw loosening, fusion rate and complications [9–12]. However, the number of studies investigating the potential correlation between cage subsidence and Hounsfield units value in TLIF was relatively limited. A cohort study by Zhao etc. proposed that lower preoperative HU values was associated with cage subsidence after TLIF with unilateral fixation [13]. In this study, we intended to compare imaging and clinical outcome differences between patients with and without cage subsidence. The efficiency in predicting cage subsidence following TLIF between two methods (HU and T score of BMD) was also evaluated and compared.

## Methods

### Study participants

The present study was a retrospective evaluation of 279 patients with lumbar degenerative diseases from April, 2016 to August, 2018. The inclusion criteria were: (1) patients aged>18 years; (2) one-level TLIF surgery with bilateral fixation of pedicle screw; (3) using the same kind of PEEK cage; (4) minimum follow-up of 12 months. The exclusion criteria were (1) spinal fracture, infection, and tumor; (2) history of spinal surgery; (3) loss of follow-up. All surgeries were performed under the instructions of standardized TLIF procedures utilizing the same type of bullet-shaped polyetheretherketone (PEEK) cage (OTWINS® lumbar cage, LIBEIER Bioengineering Institute Co., Ltd., China) by the same surgeon. Allograft (BIO-GENE® allograft, Datsing Bio-tech Co., Ltd., China) was used for better fusion when the amount of autograft bone was limited. Drainage tube was inserted and removed within 72 hours after surgery. All patients wore a hard brace for 3 months following surgery.

### Data collection and analysis

**Fig. 1:**
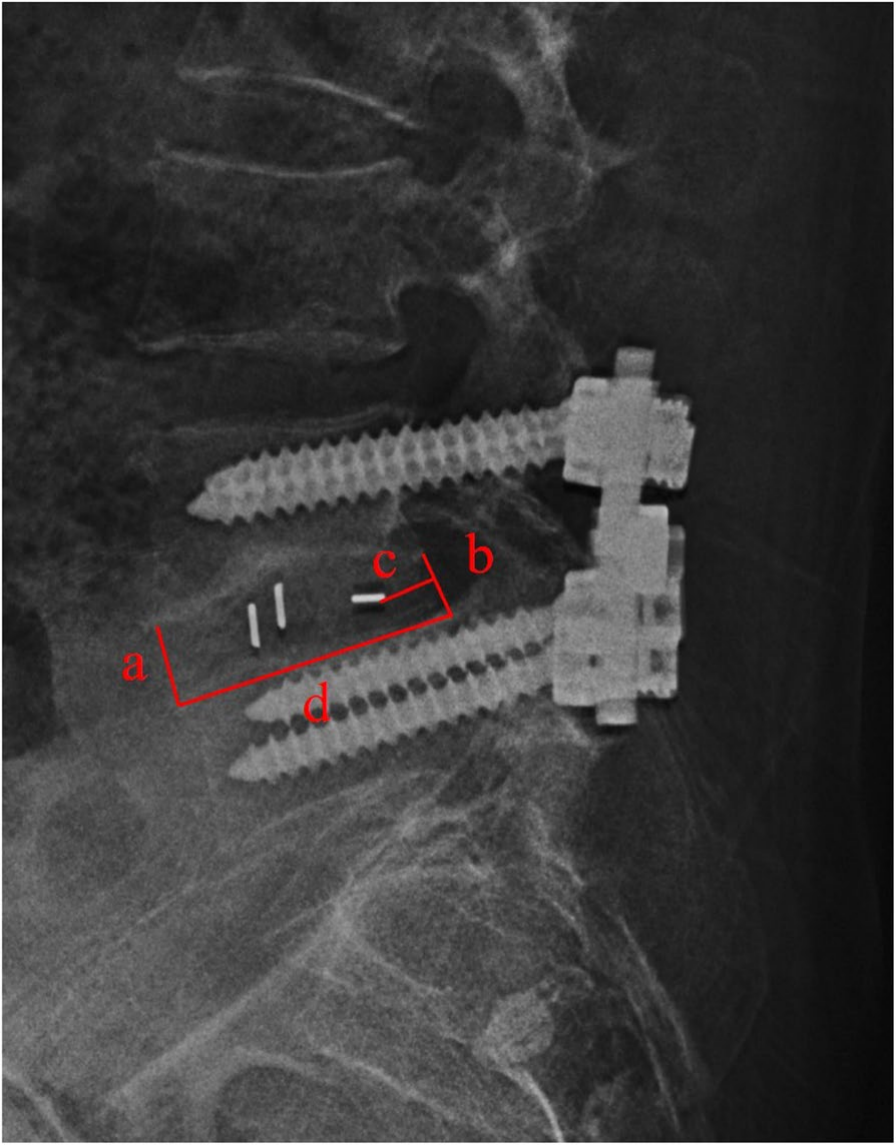
Illustration of disc height and modified Taillard index. Disc height was calculated as 2/(a + b). Modified Taillard index was calculated as c/d

General information consisted of age, sex, smoking history, diabetes mellitus, BMI and time of follow-up. Surgery-related parameters were fusion level, surgical time and blood loss. Cage-related indexes included disc height, cage position and cage height. Disc height was defined as the mean value of anterior (a) and posterior (b) regions of disc space [13]. Cage position was obtained through modified Taillard index obtained from the ratio of c/d [14]. The “c” was the distance from the posterior metallic marker of the cage to the posterior limit of the superior endplate of the inferior fused vertebra; “d” was the sagittal length of the superior endplate of the inferior fused vertebra (Fig. 1). The two indexes were measured preoperatively, postoperatively and at follow-ups. All measurements were completed through Surgimap software version 2.3 (Nemaris Inc., New York, NY, USA). Cage subsidence was defined as the loss of disc height more than 2 mm at follow-ups compared with that measured postoperatively [15]. Conditions of fusion were evaluated on the basis of flexion/extension radiographs of lumbar spine and thin-cut CT scans. Fusion criteria were: < 5° of angular motion, ≤3 mm of translation, visible bridging bone connecting the adjacent vertebral bodies, and an absence of radiolucent lines around > 50% of implant [16].

**Fig. 2:**
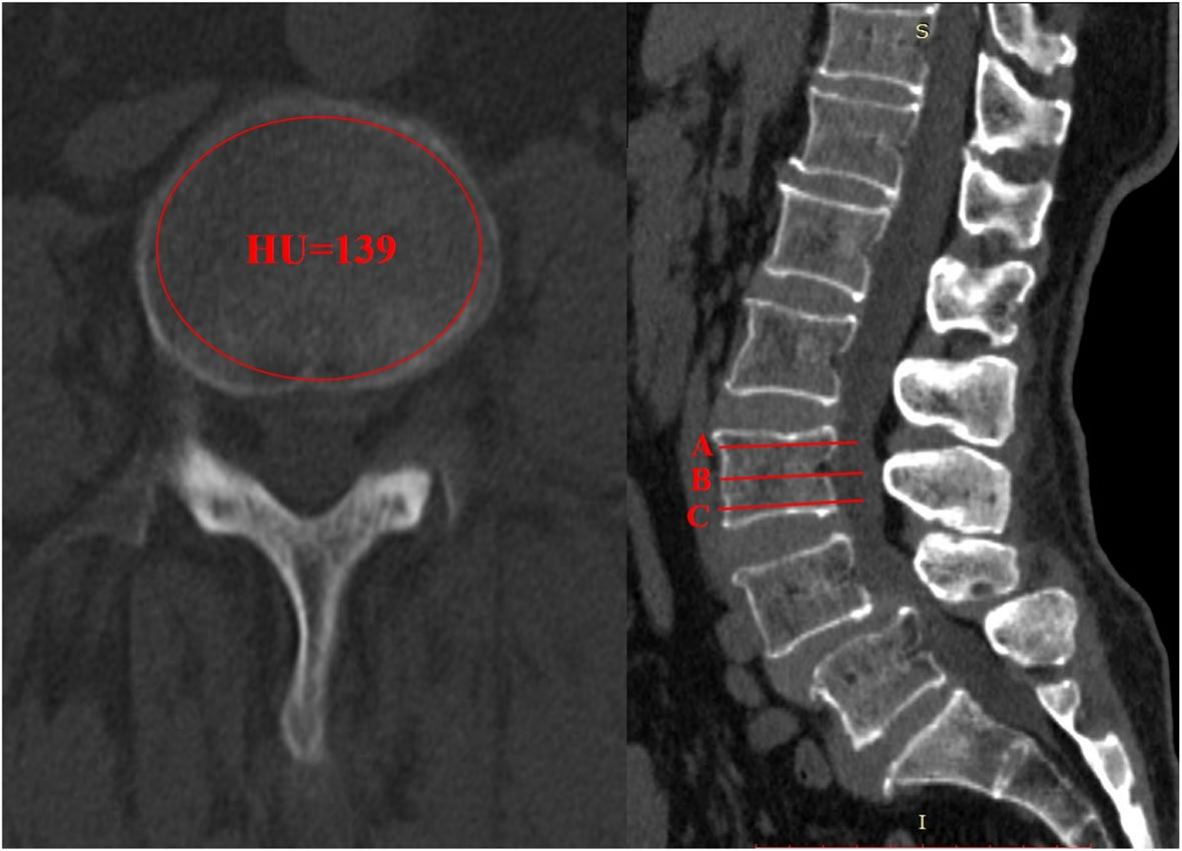
Illustration of HU measurement of one vertebra. The largest possible elliptical region of interest (ROI) over an axial level of one vertebra was drawn and HU value was obtained (left). 3 levels of each vertebral body were selected (inferior to the superior end plate, in the middle of the vertebral body, and superior to the inferior end plate) and measured (right). The mean HU value of the three levels was calculated as HU of one vertebra

Bone quality were evaluated from both T-score of DXA and Hounsfield units value of CT. Hounsfield values from L1 to L4 were assessed through CT scans utilizing Picture Archiving and Communication System (PACS). HU value was measured automatically by placing the largest possible elliptical region of interest (ROI) over an axial level of one vertebra without cortical margins. Three levels of each vertebral body were selected (inferior to the superior end plate, in the middle of the vertebral body, and superior to the inferior end plate) and mean value of the three parameters were recorded as HU value of one vertebral body (Fig. 2) [9]. HU value was recorded respectively as HU1, HU2, HU3 and HU4 from L1 to L4. HUm was defined as the mean HU value of the 4 vertebrae in one patient. DXA was performed on lumbar spinal vertebrae (L1-L4) and femoral neck. The T scores were recorded as BMD of lumbar spine (BMD-l) and BMD of femoral neck (BMD-f). Clinical outcomes including VAS score of back/leg and ODI index were obtained preoperatively, at 3 months and last follow-ups.

### Statistical analysis

All data analyses were performed using SPSS (version 19.0; IBM Corp., Armonk, NY, USA). Intra- and inter-class correlation coefficients (ICCs) were measured in imaging parameters from two independent observers unrelated with the whole surgeries. The values are expressed by mean ± standard deviation or median (25% interquartile, 75% interquartile) following normality analysis. Continuous data were compared using the independent t or Mann-Whitney U test. Categorical data were shown as (n/%) and compared using Chi-square tests. The correlations in parameters were performed utilizing Spearman tests. ROCs were compared between BMD and HU values in predicting cage subsidence. McNemar and Chi-square/ Fisher exact tests were utilized to compare sensitivity and specificity in different predicting models. AUCs were compared using Z test. A *P* <  0.05 was determined as statistical significance value.

## Results

Two hundred and seventy-nine patients (143 males and 136 females) were enrolled with mean age of 50.9 ± 8.8 years. Median of follow-up was 18 months (range from 12 to 40 months). The distributions of fusion level were 8 at L3/4, 161 at L4/5 and 110 at L5/S1. Eighty-two patients (29.4%) presented cage subsidence and were divided into CS group.

ICCs of HU values, disc height, and Modified Taillard index were all above 0.9 and could be accepted as excellent (Table 1).

**Table 1:** Intra-and inter-class correlation coefficient (ICCs) of imaging parameters

Parameters	Inter-observer (A and B)	Intra-observer (Observer A)	Intra-observer (Observer B)
HU values	0.925	0.932	0.948
Modifeid Taillard index	0.903	0.913	0.921
Preoperative disc height	0.945	0.933	0.956
Postoperative disc height	0.933	0.910	0.947
Follow-up disc height	0.901	0.923	0.932

There was no significant difference in age, sex, smoking history, diabetes mellitus, fusion level, surgical time, intraoperative blood loss, allograft usage and follow-up time between the two groups. BMI was higher in CS group. T scores of BMD-l, BMD-f and HU values were lower in CS group (Table 2).

**Table 2:** Comparison of parameters between CS group and non-CS group

Parameters	CS group (*n* = 82)	Non-CS group (*n* = 197)	P
Age (years)	51.7 ± 10.5	50.5 ± 8.1	0.351
Gender (Male)	44 (53.7)	93 (47.2)	0.359
Smoking (n, %)	25 (30.5)	40 (20.3)	0.087
Diabetes (n, %)	12 (14.6)	23 (11.7)	0.552
Fusion level (n, %)			0.452
L3/4	2 (2.4%)	6 (3.0%)	
L4/5	43 (52.4%)	118 (59.9%)	
L5/S1	37 (45.1%)	73 (37.1%)	
Follow-up (month)	18 (14, 25)	18 (15, 24)	0.626
Blood loss (ml)	153.3 ± 46.7	149.2 ± 51.7	0.523
Operation time (min)	170.9 ± 31.4	177.4 ± 30.8	0.118
Use of allograft (n, %)	4 (4.8%)	16 (8.1)	0.339
BMI (Kg/m^2^)	24.9 ± 1.8	24.3 ± 1.5	**0.02**
BMD-l	−1.1 ± 0.8	−0.2 ± 1.1	**< 0.01**
BMD-f	−2.0 (−2.3, − 1.3)	− 0.6 (− 1.3, 0.5)	**< 0.01**
HUm	116.1 ± 16.6	146.0 ± 18.7	**< 0.01**
HU1	121.2 ± 17.3	149.7 ± 20.7	**< 0.01**
HU2	114.7 ± 17.3	143.5 ± 19.9	**< 0.01**
HU3	114.7 ± 19.1	145.3 ± 19.3	**< 0.01**
HU4	113.9 ± 18.2	145.8 ± 20.2	**< 0.01**
Cage height (mm)			0.534
8	14 (17.1%)	30 (15.2%)	
10	56 (68.3%)	127 (64.5%)	
12	12 (14.6%)	40 (20.3%)	
Preoperative Disc height (mm)	9.4 ± 0.8	9.6 ± 0.9	**0.03**
Postoperative Disc height (mm)	12.7 ± 0.9	12.6 ± 0.8	0.09
Follow-up Disc height (mm)	10.8 ± 0.8	11.4 ± 0.9	**< 0.01**
Modified Taillard index (%)	31.0 ± 4.9	32.5 ± 5.1	**0.02**
Fusion rate	85.4%	92.4%	0.07
Preoperative VAS of back	5.5 (5, 6)	5.0 (5, 7)	0.27
3 m postoperative VAS of back	2 (1, 3)	2 (1, 3)	0.30
Follow-up VAS of back	2 (1, 2)	2 (1, 2)	0.08
Preoperative VAS of leg	6 (5, 7)	6 (5, 7)	0.48
3 m postoperative VAS of leg	2 (2, 3)	2 (1, 2)	**< 0.01**
Follow-up VAS of leg	2 (1, 2)	2 (1, 2)	**0.02**
Preoperative ODI	58.5 ± 13.7	58.2 ± 12.3	0.84
3 m postoperative ODI	36.7 ± 9.7	34.4 ± 8.9	0.06
Follow-up ODI	24.6 ± 6.4	22.4 ± 7.7	**0.02**

Disc height at preoperative timepoint and last follow-up in CS group was lower (9.4 ± 0.8 mm vs 9.6 ± 0.9 mm, *P* = 0.03; 10.8 ± 0.8 mm vs 11.4 ± 0.9 mm, *P* <  0.01). No differences of postoperative disc height were detected between the two groups. Modified Taillard index in non-CS group was higher than in CS group (32.5 ± 5.1 vs 31.0 ± 4.9, *P* = 0.02) (Table 2).

A total of 252 patients (90.3%) presented fusion. 12 (14.6%) in CS group and 15 (8.2%) in non-CS group were not up to fusion criteria. Fusion rates were similar between two groups (Table 2).

The rate of complications was low without statistical difference between the groups. Two cases of impaired wound healing and 2 cases of cerebrospinal fluid leakage occurred in CS group. In non-CS group, 4 cases of impaired wound healing and 3 cases of cerebrospinal fluid leakage happened. No screw-related complication including loosing or broken were found. All complications were well managed conservatively.

**Fig. 3:**
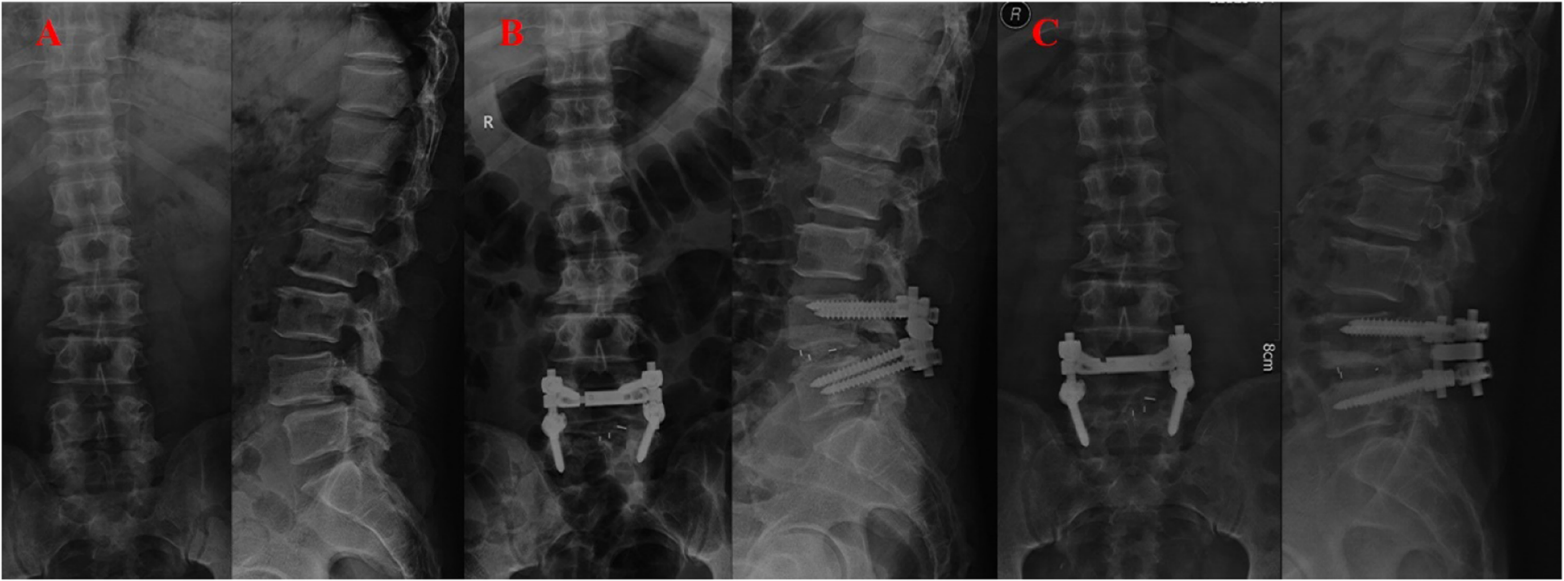
A 59-year-old gentleman underwent L4/5 TLIF with cage subsidence. Disc height was 6.2 mm preoperatively (A), 10.8 mm immediately after surgery (B), and 7.5 mm at 16 months’ follow-up (C). Pre-operative HU were 112 for HU1, 104 for HU2, 94 for HU3 and 104 for HU4. Solid fusion was not defined with invisible bridging bone connecting the adjacent vertebral bodies. ODI was 44.4% preoperatively, 8.9% at 3 months’ follow-up and 35.6% at 16-months-follow-up

VAS of back between the two groups showed no significant difference at three time points. VAS of leg was better in non-CS group at 3 months and last follow-up (*P* <  0.01; *P* = 0.02). At last follow-up, ODI index was better in non-CS group (22.4 ± 7.7 vs 24.6 ± 6.4, *P* = 0.02) (Table 2 and Fig. 3).

The correlations in different parameters were shown in Table 3. HUm was correlated strongly with BMD-l, BMD-f and BMI. Parameters with *P* < 0.05 in univariate analysis were then entered into a binary logistic regression model. BMD and Hum were correlated with the existence of cage subsidence (Tables 4, and 5).

**Table 3:** Correlations between HUm and other parameters

	HU	BMD-l	BMD-f	BMI	Preoperative disc height
BMD-l	0.467^a^				
BMD-f	0.661^a^	0.696^a^			
BMI	−0.086	0.028	−0.004		
Preoperative disc height	0.081	0.059	0.138^b^	−0.138^b^	
Modified Taillard index	0.064	0.047	0.102	− 0.079	0.115

^a^*P* < 0.01, ^b^*P* < 0.05

**Table 4:** Binary logistic regression analysis of cage subsidence (BMD-l as independent variable)

Independent variable	β	SE	P	Exp (β)	95% CI
BMI	− 0.182	0.124	0.142	0.834	0.654–1.063
BMD-l	0.649	0.196	**< 0.01**	1.913	1.304–2.807
HUm	0.073	0.01	**< 0.01**	1.076	1.054–1.098
Preoperative disc height	0.204	0.211	0.334	1.226	0.811–1.854
Modified Taillard index	0.062	0.034	0.07	1.064	0.995–1.138

**Table 5:** Binary logistic regression analysis of cage subsidence (BMD-f as independent variable)

Independent variable	β	SE	P	Exp (β)	95% CI
BMI	−0.161	0.121	0.184	0.851	0.671–1.08
BMD-f	0.469	0.223	**0.036**	1.598	1.031–2.475
HUm	0.066	0.012	**< 0.01**	1.068	1.044–1.092
Preoperative disc height	0.216	0.208	0.299	1.241	0.826–1.865
Modified Taillard index	0.057	0.034	0.093	1.059	0.991–1.131

The areas under the curve (AUC) predicting cage subsidence were: 0.754 for BMD-l (sensitivity 86.6%, specificity 51.8%), 0.821 for BMD-f (sensitivity 76.8%, specificity 73.6%) and 0.89 for HUm (sensitivity 92.7%, specificity 72.6%) (Table 6). Pairwise comparisons of AUCs among HUm, BMD-l and BMD-f were conducted. Between HUm and BMD-l, AUC for HUm was larger (Z = 3.83, *P* < 0.01). McNemar test showed significant difference for an overall test for both sensitivities and specificities (χ^2^ = 22.2, *P* < 0.01). No statistical difference was detected between sensitivities (*P* = 0.182) while specificity for HUm was higher (χ^2^ = 11.7, *P* < 0.01). Between HUm and BMD-f, AUC for HUm was larger (Z = 2.01, *P* = 0.02). McNemar test showed significant difference for an overall test for both sensitivities and specificities (χ^2^ = 11.8, P < 0.01). No statistical difference was detected between specificities (*P* = 0.551) while sensitivity for HUm was higher (χ^2^ = 9.6, *P* < 0.01). Between BMD-l and BMD-f, no significant difference was detected (Z = 1.63, *P* = 0.052). No statistical difference was detected between two sensitivities (χ2 = 3.1, *P* = 0.077) while specificity for BMD-f was higher than that for BMD-l (χ2 = 30.25, P < 0.01).

**Table 6:** Results of receiver operating characteristic curves in predicting cage subsidence using different parameters

Parameters	Area under the curve (AUC)	SE	P	95% CI
BMD-l	0.754	0.03	**< 0.01**	0.694–0.813
BMD-f	0.821	0.028	**< 0.01**	0.766–0.876
HUm	0.890	0.019	**< 0.01**	0.853–0.926
HU1	0.850	0.023	**< 0.01**	0.805–0.895
HU2	0.862	0.022	**< 0.01**	0.818–0.906
HU3	0.870	0.022	**< 0.01**	0.828–0.912
HU4	0.884	0.019	**< 0.01**	0.846–0.922

AUC for HU4 was larger than BMD-f (Z = 1.85, *P* = 0.03) and close to HUm (Z = 0.22, *P* = 0.481). No significant differences were found between BMD-f and HU value of the other three vertebrae for AUC.

## Discussion

In present study, the incidence of cage subsidence was 29.4%, and HUm could be a more effective predictor of postoperative cage subsidence with larger AUC compared with BMD-l/BMD-f.

Lumbar interbody fusion was widely performed in managing lumbar degenerative diseases. Stable fusion and restoration of disc height contributed to satisfactory clinical results. However, cage subsidence may appear following lumbar fusion surgeries and lead to unsatisfactory results because of decrease of disc height and restenosis of the foramen regions [17]. Some previous literatures reported no negative impact of cage subsidence on clinical outcomes [5, 18, 19]. While recent studies pointed out that in patients with cage subsidence, the improvement of ODI index was worse than those without cage subsidence [6]. Overall, the relationship between clinical outcomes and cage subsidence remained controversial.

From the results, we found that improvement of clinical outcomes in non-CS group was better. A possible explanation was that in our study and from our experience, most of the patients were implanted cage less than 12 mm. The patients may be more sensitive to the loss of disc height as the degree of height restoration was relatively lower. We seldom selected cage with height of 14 mm or higher, for cage with large height may result in more preparation of disc space, which increase the risk of endplate injury. Bach et.al summarized disc height in healthy individuals and proposed that a cage greater than 10 mm cage height will result in excessive restoration and potential risks of complications [20]. An obvious characteristic of our patients was that the course of disease was long (mean 4.4 years), which led to severe degenerative conditions of lumbar spine and the disc height decreased obviously. We believed specific conditions of the patients should be considered when selecting appropriate cage height.

The rate of cage subsidence after TLIF ranged around 35% [21]. Our findings went along with previous results at a rate of 29.4%.

A number of factors may contribute to cage subsidence following lumbar spine surgeries. Patient-related parameters consisted of age, gender, BMI, and bone quality [22]. Surgery-related factors including cage height, size and position were also reported. Cage position was considered as an important factor. Several methods have been reported to describe the position. Landham et.al utilized center point ratio (CPR) and posterior gap ratio (PGR) to illustrate cage position and the two parameters were correlated with gain of lumbar lordosis [23]. More anteriorly located cage was recommended to restore lumbar lordosis more effectively and avoid cage subsidence [6, 24]. In our study, in consideration of the design of cage, the position in CS group was determined by modified Taillard index, which was similar with PGR. A different point should be noted that the posterior metallic marker instead of border of cage was selected to draw “c” line, as the cage template we utilized was non-visualized through X-ray [14]. Still, the results confirmed that cage was located more anterior in non-CS group.

The correlation between bone quality and cage subsidence has been assessed in some studies. Choi et.al compared HU value and DXA to assess bone quality in 80 non-lumbar degenerative and 30 lumbar degenerative patients. There was a strong correlation between HU value and T-score. Real bone quality may be overestimated in degenerative patients, for the osteophytes could increase the value of T-score [25]. Ullrich et.al investigated the HU value in 81 patients underwent posterior-anterior stabilization because of thoracolumbar spine fractures and found HU value was strongly correlated with cage subsidence and additional treatment strategies should be considered in patients with a HU value less than 180 [26]. A recent study by Wang et al. analyzed the correlation between cage subsidence in ACDF and HU value in cervical spine. They proposed there was a negative correlation between HU value and segmental height loss at the surgical level [27]. Overall, the value of HU in lumbar spine surgeries with fusion has not been fully investigated. In our study, we measured the HU values from L1 to L4, which were consistent with the reports of BMD. A mean value of the 4 vertebrae was recorded as HUm. The results showed HU value was strongly correlated with T score of BMD of lumbar spine, which was similar with previous reports [28]. Besides, BMD from femoral neck was also taken into account. Spearman analysis confirmed that BMD-f still correlated with HU and the r value (0.661) seemed to be higher than that of BMD-l. Osteophyte formation and bone sclerosis may increase BMD and make bone quality overestimated. We concluded BMD-f provided a more authentic reflection of bone quality compared with BMD-l.

The differences of BMD, BMI, cage position, age and HU values were significant between the two groups in our study. Binary logistic regression analysis showed BMD and HU were risk factors for cage subsidence. Cage position failed to reach a statistical significance. A potential explanation was that the surgeries were performed by the same surgeon, the procedures and intraoperative habits were consistent, which means the variation of cage position was relatively small. Different predicting models were performed. AUC of BMD-f seemed to be larger than BMD-l, while statistical difference was not achieved (*P* = 0.052). However, the specificity was higher in BMD-f than that of BMD-l. It could be accepted that BMD-f performed better than BMD-l in predicting cage subsidence. Compared with BMD, HUm could be a more effective predicting parameter with larger AUC. HUm was calculated as the mean value of 4 vertebrae, which was time-consuming with 12 times measurements required. To investigate and possibility of simplifying procedures, we separately analyzed the value per vertebra in predicting cage subsidence. The results showed HU4 alone may act as a substitute for HUm with a similar AUC. This could be easily applied in clinical experience as three times of measurements were enough. The results above indicated that HU values could be utilized as an easily obtained and effective value in predicting cage subsidence. CT scans were essential and regularly examined preoperatively at our center. Considering the cost of treatment, HU value could be regarded as an important parameter in surgical plan. Zhao et.al proposed that HU measurement may be used as a predictor of cage subsidence after unilateral fixation [13]. In our study, a larger sample (279 VS 36) was evaluated with bilateral fixation. Although unilateral fixation may achieve satisfactory clinical results in lumbar spinal fusion surgeries in some literatures [29, 30], bilateral fixation was more widely accepted and utilized. Therefore, the results of the study could be a more practical guidance in clinical experience.

Fusion rate was also taken into consideration. Numerous methods have been proposed to assess conditions of fusion. Gruskay et.al analyzed different ways in evaluating fusion and concluded that thin-cut CT and dynamic plain films should be regarded as imaging modalities [31]. Sugiyama et.al proposed that neither plain static nor dynamic radiographs were able to evaluate fusion outcome accurately compared with CT-based assessment [32]. In our center, all patients at one-year follow-up or longer received thin-cut CT scans and dynamic films were obtained to assess fusion condition regularly. Thus, we enrolled the two methods together to assess fusion rate. Fusion rate in CS group was lower but achieved no significance. The possible correlation between cage subsidence and fusion rate required a more detailed exploration.

This study has several limitations. Firstly, the cohort was relatively small. All patients received one level TLIF below L2/3 level and the same type of PEEK cage, which might mean selection bias. A second limitation is that 66 patients (23.7%) received BMD detection of femoral neck preoperatively. The other BMD-f values were obtained at follow-ups. Although no significant difference was found between preoperative values and values at follow-ups in the 66 patients, a more accurate comparison could be implemented using complete preoperative parameters. A third limitation is that mean value of BMI was around 25Kg/m^2^ and the whole cohort should be regarded as normal population. The potential difference in overweight or obese sample is worth further studying. Lastly, the follow-ups in this study were relatively short. A longer and prospective investigation is needed in the future. Lastly, the retrospective study proposed HU at L4 may act as a convenient predictor of cage subsidence with fewer measurements compared with HUm. A prospective study with larger sample is essential to validate whether this conclusion still hold.

## Conclusions

Cage subsidence may indicate worse clinical outcomes. HU value correlates strongly with BMD of lumbar spine/femoral neck and BMI. HU value could be a more effective predictor of lumbar cage subsidence compared with T-score of DXA in TLIF. Preoperative HU value measurement could be considered as a tool in evaluating bone quality more comprehensively.

## Data Availability

The datasets generated and/or analyzed during the current study are not publicly available but are available from the corresponding author on reasonable request.
